# Human Brain Activity Patterns beyond the Isoelectric Line of Extreme Deep Coma

**DOI:** 10.1371/journal.pone.0075257

**Published:** 2013-09-18

**Authors:** Daniel Kroeger, Bogdan Florea, Florin Amzica

**Affiliations:** 1 Department of Stomatology, School of Dentistry, Université de Montreal, Montreal, Canada; 2 Medical Centre Regina Maria, Cluj-Napoca, Romania; University of Alberta, Canada

## Abstract

The electroencephalogram (EEG) reflects brain electrical activity. A flat (isoelectric) EEG, which is usually recorded during very deep coma, is considered to be a turning point between a living brain and a deceased brain. Therefore the isoelectric EEG constitutes, together with evidence of irreversible structural brain damage, one of the criteria for the assessment of brain death. In this study we use EEG recordings for humans on the one hand, and on the other hand double simultaneous intracellular recordings in the cortex and hippocampus, combined with EEG, in cats. They serve to demonstrate that a novel brain phenomenon is observable in both humans and animals during coma that is deeper than the one reflected by the isoelectric EEG, and that this state is characterized by brain activity generated within the hippocampal formation. This new state was induced either by medication applied to postanoxic coma (in human) or by application of high doses of anesthesia (isoflurane in animals) leading to an EEG activity of quasi-rhythmic sharp waves which henceforth we propose to call ν-complexes (Nu-complexes). Using simultaneous intracellular recordings *in vivo* in the cortex and hippocampus (especially in the CA3 region) we demonstrate that ν-complexes arise in the hippocampus and are subsequently transmitted to the cortex. The genesis of a hippocampal ν-complex depends upon another hippocampal activity, known as ripple activity, which is not overtly detectable at the cortical level. Based on our observations, we propose a scenario of how self-oscillations in hippocampal neurons can lead to a whole brain phenomenon during coma.

## Introduction

Regardless of the underlying causes, coma is a state during which the brain reaches a low level of neuronal activity and metabolism. Possible etiologies range from safe and fully reversible pharmacological interventions (such as general anesthesia) to severe, irreversible brain damage. There is virtually no systematic investigation of the cerebral cellular mechanisms at work during coma and attempts to compare pathological and pharmacological etiologies are scarce. It is therefore no surprise that the outcome from coma is often predicted on a statistical basis. Recent results from our laboratory have highlighted two unexpected findings. First, we have shown that coma induced by a variety of anesthetics presents a time-frame during which the cortex is in a hyperexcitable state that is responsible for the genesis of the burst-suppression (BS) pattern [1]. Second, we reported evidence that isoflurane-induced BS opens the blood-brain barrier [2].

BS was first described by Swank and Watson [3]. Its main feature at the EEG level consists of quasi-periodical bursts of bilateral high-amplitude slow waves (mainly <15 Hz) separated by low-amplitude or absent activity lasting from a few seconds to minutes [4]. The first cellular correlates of BS were revealed by Steriade and colleagues [5], demonstrating that EEG bursts were associated with excitatory activities in cortical neurons, while suppression phases were paralleled by absence of cortical network interactions. The same study showed that thalamic neurons displayed spontaneous discharges throughout BS with no apparent correlation to the two phases of BS. Overall, BS was mainly investigated as a prognostic tool during coma (reviewed in [6]).

The common clinical correlate of coma is loss of consciousness and low or absent responsiveness [4]. Although the initial stages (I–II) of coma are comparable to deep sleep [7], deep coma (stages III-IV) corresponds to more profound alterations of brain states, observable at the electroencephalographic (EEG) level [8], [9]. Deepening of the coma beyond the BS stage leads to a flat EEG called isoelectric line, which is presumed to be associated with silenced activity in cortical neurons. Such an EEG pattern is considered to be one of the limit points in establishing brain death and in particular clinical conditions it is accepted as the only criterion [10].

The activity of subcortical neurons (e.g. thalamic, hippocampal) has not been studied during EEG isoelectric line, but it might be hypothesized both from the situation encountered during the suppression phase of the BS pattern [5] and from recordings in isolated preparations (such as *in vitro*), that a rudiment of oscillatory activity might persist in subcortical neurons. Whether this activity can become synchronized and re-emerge at the cortical level is so far unknown.

The results presented here challenge the common wisdom that the isoelectric line is always associated with absent cerebral activity, and demonstrate that the isoelectric line is not necessarily one of the ultimate signs of a dying brain. We show that if cerebral neurons survive through the deepening of coma, then network activity can revive during deeper coma than the one accompanying the EEG isoelectric line by the change in the balance of hippocampal-neocortical interactions.

## Materials and Methods

All experimental procedures were performed according to NIH guiding principles and were also approved by the committee for animal care of the university (Comité pour la protection des animaux de l′Université Laval, CPAUL). As for the human recording, written consent was obtained from the family in order to use the recordings performed during his treatment for publication and no experimental paradigm was implemented on the patient. The Committee for Ethics of the Unirea (Regina Maria) Medical Centre approved of the use of the recordings for publishing purposes.

Experiments were performed on twenty-six cats (2.5–4.5 kg) of both sexes. The surgical procedures were described in detail elsewhere [1]. After the initial dose of ketamine-xylazine (15 mg/kg and 3 mg/kg, respectively), animals were paralyzed (gallamine triethiodide), and anesthesia was switched to isoflurane (1.3–1.5%). After a stable baseline recording containing continuous slow and ample EEG waves, the isoflurane was increased to 4% in order to induce νC patterns. During the experiments vital parameters were continuously monitored and maintained within physiological limits: body temperature (37±0.2°C), expired CO_2_ (3.7±0.2%), respiration rate (20–30 strokes/min) and heart rate (<110 beats/min). Through craniotomy we exposed the suprasylvian gyrus, where intracellular pipettes and field electrodes were lowered into the cortex and in the hippocampus using stereotaxic coordinates. The hippocampal pipettes were lowered through the suprasylvian gyrus and lateral ventricles aiming at the CA3 region of the hippocampus (AP+5; L+5.5; H+7.5 - in parallel to the midline). Neuroanatomical evidence for the placement of the recording electrodes was obtained for a few cells by staining neurons with intracellular injection of Lucifer yellow (3%, Invitrogen; dissolved in a 1 M LiCl_2_ solution, Sigma). The intracellular filling was achieved by applying hyperpolarizing pulses (500 ms at 1 Hz) of 1–3 nA for 10–15 min. At the end of the experiments the brains were perfused with 4% paraformaldehyde and stored in paraformaldehyde and 30% sucrose for 2–3 days. Sections of 75 µm were taken and cells were revealed using a Zeiss confocal laser-scanning microscope (LSM 5 PASCAL, Zeiss, Oberkochen, Germany).

Human EEG recordings were performed in monopolar configuration with the reference placed on the earlobes and crosslinked. Due to the supine position of the patient occipital electrodes could not be placed in the 10–20 classical configuration. Instead they were positioned more laterally, closer to the temporal ones. Filtering was set for all channels between 0.1 and 300 Hz, with the notch filter on.

The analysis was performed with software from WaveMetrics Inc. (Igor software, Oregon, USA), and relies on time relationships between the recorded voltage time series. Most of the results are presented as event- or spike-triggered averages. They were obtained after extracting from the intracellular or extracellular field/EEG potentials sweeps synchronized with the triggering event (action potentials or extreme of a particular waveform) and averaging them. This provided the statistical evidence for interactions between the discharge in the reference channel and the field/EEG potential variations of the target channel. Cross-correlograms were derived in order to detect time relationships between events of similar origin recorded from different channels. The procedure consisted in detecting events in two simultaneously recorded channels. The events in one channel were kept as time reference (time zero), and the time lags of the events in the other channel with respect to each event in the reference channel were plotted in a histogram.

## Results

### EEG Phenomena in Comatose Patients

**Figure 1:**
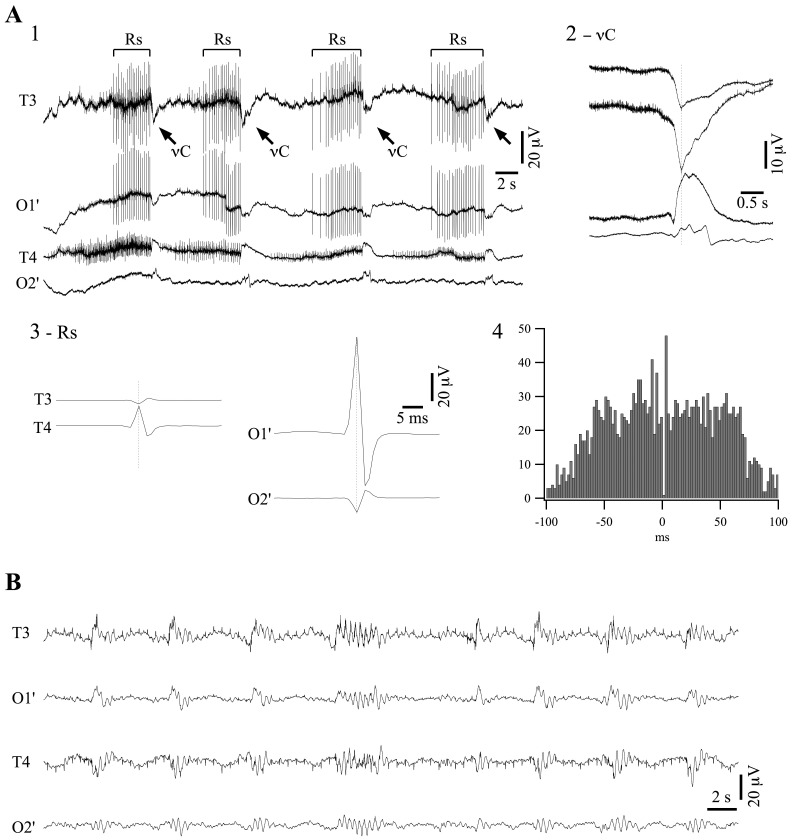
EEG recording of a patient in deep coma. (**A1**) Typical pattern of EEG recorded from temporal (T3 and T4) and modified occipital (O1′ and O2′) derivations. The pattern is dominated by ripples (Rs; *bracket lines*) and ν-complexes (νCs; *oblique arrows*). The recording also contains cardiac contamination, as often in comatose patients. (**A2**) Averaged νCs (*n* = 350). The negative peak of νCs in the O1′ derivation was detected and sweeps of 4 s around that time marker were extracted from each derivation and averaged. (**A3**) Averaged ripples (*n* = 5000). *Left panel*: Positive peak detection of Rs in the T4 derivation and averaging of all signals reveals no potentials in the occipital leads (not shown) and a reverted, less ample, ripple in T3. *Right panel*: similarly, positive peak detection in the O1′ derivation and averaging of all signals shows only a small and reverted ripple in O2′. (**A4**) Cross-correlogram between ripples in T4 and O1′ shows no time relationship. (**B**) Same patient, after removing antiepileptic drugs (carbamazepine, diazepam and thiopental) displays a pattern of burst-suppression. Here again, contamination of the EEG by the electrocardiogram, visible especially in the temporal leads. In this and following figures, all potentials are depicted with positivity upwards.

One of the main challenges in clinical practice is the fact that the interpretation of the EEG of a comatose patient is often hindered by the lack of information concerning the dynamic progression of the patient’s state. Such an exceptional case was represented by the EEG pattern displayed in Fig. 1. The patient was admitted in the emergency service after cardiorespiratory arrest and successful resuscitation. By the time of his arrival at the hospital he was unconscious and presented quasiperiodic generalized convulsions. He was administered antiepileptic medication (carbamazepine 3 times 200 mg per day, diazepam 3 times 10 mg per day, and thiopental i.v. 175 µg/kg/h) and this reduced the convulsions to quasiperiodic jerks.

EEG recordings performed at this time showed a pattern like the one depicted in Fig. 1A1. Most unusually, it was dominated by bursts of rhythmic spike-like discharges (average frequency of 5.8±0.9 Hz; values are given as mean ± standard deviation throughout the paper), somewhat akin in shape and frequency to hippocampal ripple events (Rs), with each burst lasting for several seconds. They seemed to originate independently in several foci. We here give the example of those recorded in the T4 derivation (Fig. 1A3, left panel) and those of O1′ (Fig. 1A3, right panel). Each trace represents the average of 5000 ripple events. Ripples originating at one location were also recorded by contralateral electrodes with reversed polarity. The simultaneity of the two waveforms and their opposed polarity has been encountered in situations with an interposed dipole, although a definite conclusion is difficult to draw at this point. Moreover, there was no time relationship between ripples generated at different recording sites (Fig. 1A4) indicating that these events were not propagating through cerebral circuits but rather being generated by independent oscillatory structures and recorded by our electrodes through volume transmission.

Other peculiar waveforms present during these recordings were ample and slower deflections occurring simultaneous with the motor jerks of the patient (indicated by oblique arrows in Fig. 1A1). For reasons that will become obvious further, we call this waveform ν-complex (νC). Its aspect is further detailed in Fig. 1A2 where averaged νCs (*n = *350) are characterized by consistent polarity in any of the recorded leads (positive on the right, negative on the left). νCs appeared pseudo-rhythmically every ∼10 s (average time interval of 9.3±2.7 s; *n* = 350).

After 6 days spent in this state, the antiepileptic medication was discontinued and, after passing through a period of isoelectric activity, the EEG assumed a pattern of burst-suppression (Fig. 1B) with frequent stereotyped burst shapes as described elsewhere [1]. This latter pattern is usually present during deep coma [4], and in this case was achieved by removing pharmacological agents such as thiopental reputed to deepen coma. Therefore we submit that under antiepileptic medication the patient was in a deeper state of coma. Moreover this state was accompanied by novel EEG patterns that are not described in the literature. We therefore planned a series of experiments with laboratory animals in order to elucidate the cellular mechanisms underlying this behavior.

### EEG Phenomena in Comatose Animals

**Figure 2:**
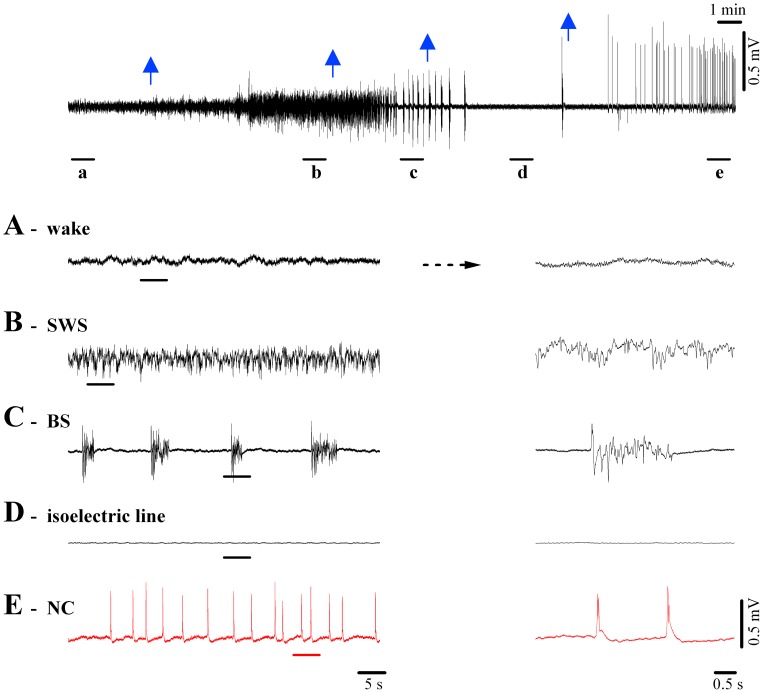
EEG patterns during wakefulness and diverse degrees of loss of consciousness. Cat EEG recording during application of various concentrations of isoflurane. Whilst the top-most panel depicts the complete sequence with arrows indicating the applications of increasing anesthesia, the underlined epochs are expanded below. (**A**) Undrugged preparation displaying low amplitude, fast (mostly >15 Hz) EEG. (**B**) Slow-wave sleep-like (SWS) pattern after 1% isoflurane, characterized by higher amplitude slow waves dominated by delta oscillations (<4 Hz). (**C**) Burst-suppression (BS) induced with 2% isoflurane showing alternating sequences of isoelectric line and bursting episodes. The latter are very similar to SWS patterns (see detail at right). (**D**) A further increase in the isoflurane concentration (3%) establishes a stable isoelectric line portraying the absence of phasic events. Only very low-amplitude activities can be observed at high gain. (**E**) Isoflurane at 4% elicits a revival of quasi-rhythmic spiky potentials of high amplitude, which we propose to call ν-complexes (or Nu-complexes; νC).

Fig. 2A–D summarizes the essential EEG features accompanying the deepening of coma, as obtained with progressive doses of the anesthetic isoflurane applied in cats. Wakefulness (Fig. 2A) and sleep-like oscillations (Fig. 2B) have been extensively studied and their cellular and ionic substrates are well understood (for review, see [11]). Briefly, the EEG of wakefulness displays a relatively low amplitude and contains fast waves with a frequency spectrum generally above 15 Hz. Slow-wave sleep (SWS)-like patterns were induced with an initial dose of isoflurane of 1% (first blue arrow in Fig. 2). The EEG pattern of SWS is dominated by slow oscillations (<1 Hz) [12] intermingled with other rhythms such as delta (<4 Hz) and spindles (7–15 Hz) [11].

The BS pattern shown in Fig. 2C, first described by Swank and Watson [3], is associated with deep coma and generally develops during anesthesia (in our case, isoflurane at 2% applied at the 2^nd^ arrow in Fig. 2), hypoxia, cardiac arrest, drug-related intoxications, childhood encephalopaties, hypothermia, etc. [13], [14], [15], [16], [17], [18]. The neurophysiological mechanisms responsible for generating BS patterns remained elusive for many years. Recently we have shown that BS induced with various anesthetics (isoflurane, propofol, barbiturates) is associated with a state of cortical hyperexcitability in that the bursts of the BS pattern can be triggered by subliminal stimuli reaching the hyperexcitable cortex [1].

Fig. 2D depicts the EEG isoelectric line following increased doses of isoflurane anesthesia (3% at the 3^rd^ arrow in Fig. 2), reflecting a further deepening of the coma. Such EEG patterns are considered to portray “electrocerebral silence” and are one of the hallmarks in establishing brain death. Therefore, in clinical conditions, the presence of a prolonged isoelectric line in a comatose patient is, among others, one of the determinants of a fatal diagnosis [10].

The novelty reported in this study is that through an application of anesthetics beyond what is required for the induction of the isoelectric line (isoflurane >3.5%) we obtained a re-vitalization of activity in the brain, reflected by the EEG (Fig. 2E). This activity was characterized by sharp EEG waves occurring quasi-rhythmically at frequencies below 1 Hz. Since this EEG wave has been unknown until now, we propose to call it ν-complex (Nu-complex, νC), in deference to EEG tradition and its resemblance to the Greek letter “ν”. Given that such activity patterns have not been reported before, essential questions as to the site of genesis and underlying cellular mechanisms need answering.

### Hippocampal Activities – Recordings of ν-Complexes in Cats

**Figure 3:**
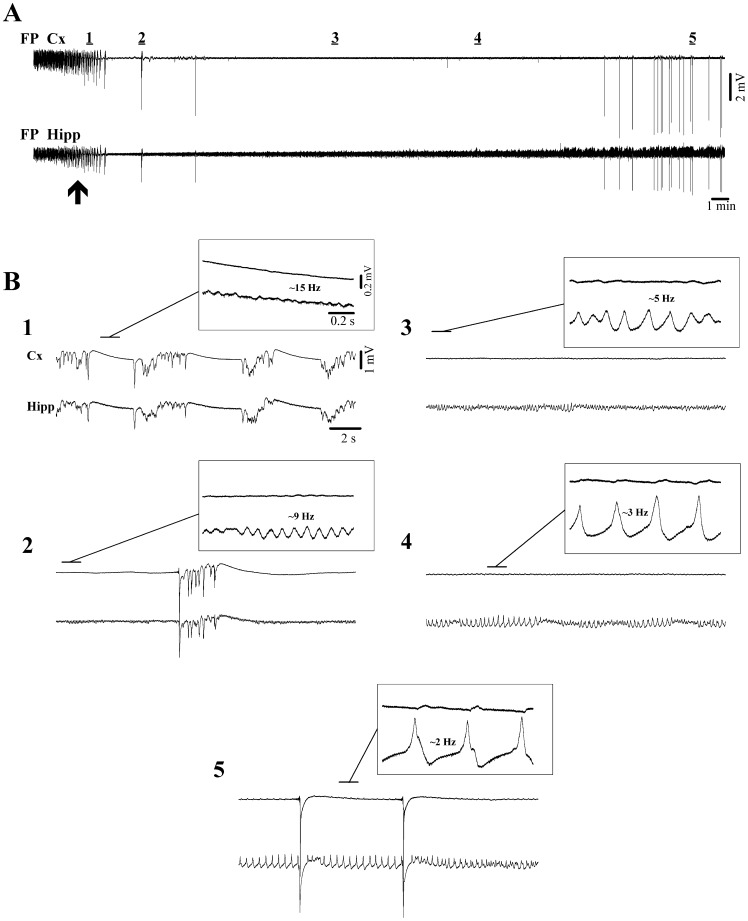
Isoflurane induction of deep coma, including the EEG isoelectric line and νC state. (**A**) Continuous recordings of intracortical field potentials (FP Cx) together with hippocampal field potentials (FP Hipp) first under lower levels of isoflurane (2%), which is then switched to 4% (at arrow) leading to a gradual suppression of bursting activity and eventually to isoelectric line. The 5 periods underlined by horizontal bars are expanded in (**B**). (**B1**) The magnification shows ripple activity in the hippocampus at ∼15 Hz during transient suppressions. (**B2**) Isolated burst surrounded by ripple activities in the hippocampus (∼9 Hz). (**B3**) & (**B4**) Continuous isoelectric line displaying progressively slower and ampler ripples in the hippocampal field potential. (**B5**) νC state characterized by ample delta ripples in the hippocampal trace interrupted occasionally by high amplitude νCs. Note that in all insets the overt expression of ripple activities in the cortex is absent.

At the EEG level, νCs were characterized by amplitudes of 1.3 mV (±0.2, *n* = 70) and duration of 129 ms (±25). Since νCs were clearly expressed in the EEG we assumed that they would primarily reflect cortical activities. Furthermore, our field potential (FP) recordings performed in all cortical areas (primary as well as associative ones) also displayed high amplitude νCs (3.4±0.4 mV) (Fig. 3A, upper trace). These FP events consisted almost exclusively of negative potentials in deep cortical layers (Fig. 3B5, upper trace), reflecting massive and synchronous cellular excitation. It became apparent from these recordings that νC-activity shared features with exclusively hippocampal phenomena such as the sharp waves during resting wakefulness and slow-wave sleep [19], and slow intrinsic spikes under urethane anesthesia [20]. Additional similarities were observed between νCs and less hippocampal-specific patterns such as epileptic interictal spikes [21]. Whether or not νCs were also present in the hippocampus or other subcortical structures was investigated with double field potential recordings. Virtually all tested structures (thalamus, basal forebrain, brainstem and hippocampus) displayed activities time-related with EEG νCs (not shown). However, among all subcortical structures, hippocampal νCs exhibited by far the greatest amplitude. Moreover, νCs from all other structures displayed a time-lag trailing hippocampal νC events by at least 10 ms, suggesting that the hippocampus might be the key structure for generating νCs. In more detail, measuring the time lags between the peaks of νCs recorded as field potentials we obtained the following values: basal forebrain was delayed with respect to the hippocampus by 14.2±3.7 ms (*n* = 240 νCs, data pooled from 3 animals), brainstem was delayed by 18.4±5.1 ms (*n* = 240, 3 animals), and thalamus (lateral pulvinar nucleus) was delayed by 9.8±4.6 ms (*n* = 240, from 3 animals).

Our simultaneous cortical and hippocampal field potential recordings also emphasized another activity exclusively present in this subcortical structure: faster ripple oscillations that were observable even during the cortical isoelectric line (Fig. 3). Indeed, these ripple oscillations were a constant feature starting with the state of BS (Fig. 3B1) and continuing throughout the transition to the EEG isoelectric line (Fig. 3B2–4) and νC state (Fig. 3B5). In all recordings of progressions from BS to νC state (*n* = 14) hippocampal ripple frequency slowed down continuously starting in the low beta range (15.8±0·9 Hz) during BS and ending within the delta range (2.2±0·7 Hz). In parallel with the slowing of the oscillatory frequency we noted an increase in the amplitude of the ripples by an average factor of 10, suggesting a progressive increase in the synchronization within hippocampal networks during ripple activity. Interestingly, νCs appeared to reset the amplitude scale for ripples (Fig. 3B5) in that the first event after a νC was dramatically smaller, whilst subsequent ripples displayed successively larger amplitudes. Although ripple activity was not overtly visible in cortical recordings we noticed that at higher amplifications and with the benefit of simultaneous hippocampal field potentials, one could distinguish coherent phasic potentials even in the cortical field potential (Fig. 3B3–5). This observation raised the question as to whether such FP events portray true intracellular oscillations by cortical neurons, or are merely the reflection of hippocampal dipoles.

### Cellular Correlates of the νC State

**Figure 4:**
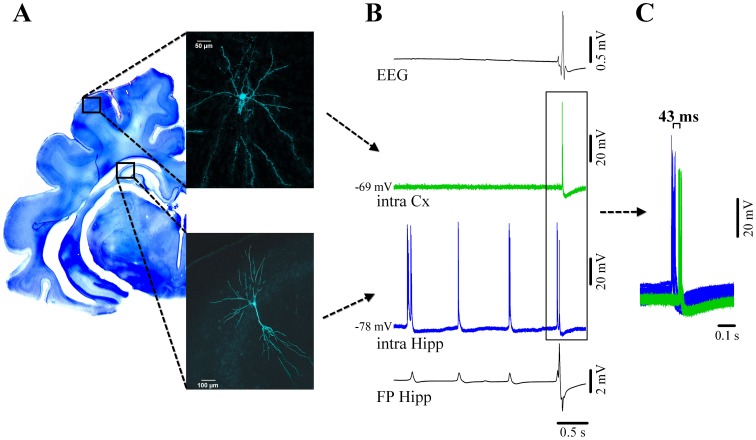
Cortical and hippocampal neuronal activity during νC state. (**A**) Neocortical pyramidal neuron from suprasylvian area 5 (above) and simultaneously recorded pyramidal CA3 hippocampal neuron filled with Lucifer Yellow and reconstructed with confocal microscopy. Their respective locations are schematically indicated on a Nissl-stained coronal section of the brain. (**B**) From top to bottom: simultaneous recording of the EEG, intracellular cortical neuron (green), intracellular hippocampal neuron (blue) and adjacent hippocampal field potential (FP). Both hippocampal traces indicate the presence of two types of activities: delta ripples at about 1 Hz (small amplitude positive potentials in the FP, accompanied by bursts of action potentials in the nearby neuron), and a νC (high amplitude spiky multiphasic potential in the FP, which is paralleled by neuronal discharge). The EEG displays a continuous isoelectric line during hippocampal ripples but displays the νC during which the cortical neuron discharges bursts of action potentials. Delta ripples are not expressed in the neocortex. (**C**) Time relationship between neuronal discharges for νC events indicating that the hippocampal discharges consistently precede the neocortical ones.

Through simultaneous intracellular recordings in the hippocampus and cortex (Fig. 4) we could determine that (a) ripple activity was absent in cortical neurons but present in hippocampal cells (Fig. 4B), and (b) intracellular νCs spiking occurred first in hippocampal neurons and was then relayed to cortical cells with an average delay of 43 ms (±4.2) (Fig. 4C). Of the 55 neurons recorded, we filled 8 cells with Lucifer Yellow to ascertain the location and morphological features, which allowed subsequent reconstruction with the use of a confocal microscope (Fig. 4A). Reconstructed hippocampal cells could be identified as CA3, CA2 and CA1 pyramidal neurons (*n* = 5), whilst pyramidal shaped and nonpyramidal shaped neurons (aspiny cells) were recovered following cortical recordings (*n* = 3). All cortical cells displayed steady membrane potentials in-between νCs and did not show any activities related to ripples (Fig. 4B). In all recorded cortical neurons (*n* = 26) EEG νCs were associated with depolarizing membrane potentials crowned by bursts of action potentials (on average 2.6 action potentials/νC). We suggest that the depolarizing potentials underlying the action potentials are triggered by EPSPs because they increase their amplitude with steady hyperpolarization of the membrane potential (not shown). At the same time, it is unlikely that IPSPs contribute to the shaping of these events since, at lesser isoflurane concentrations than the ones used here cortical inhibition is largely suppressed [22].

Ripple events were exclusively recorded in hippocampal CA3, CA2, and CA1 pyramidal cells (*n* = 39 pyramidal neurons). Ripples in the delta frequency range with depolarizing bursting activities in hippocampal neurons (on average 3.1 spikes/ripple) were time locked with local field potential events suggesting regional synchrony.

**Figure 5:**
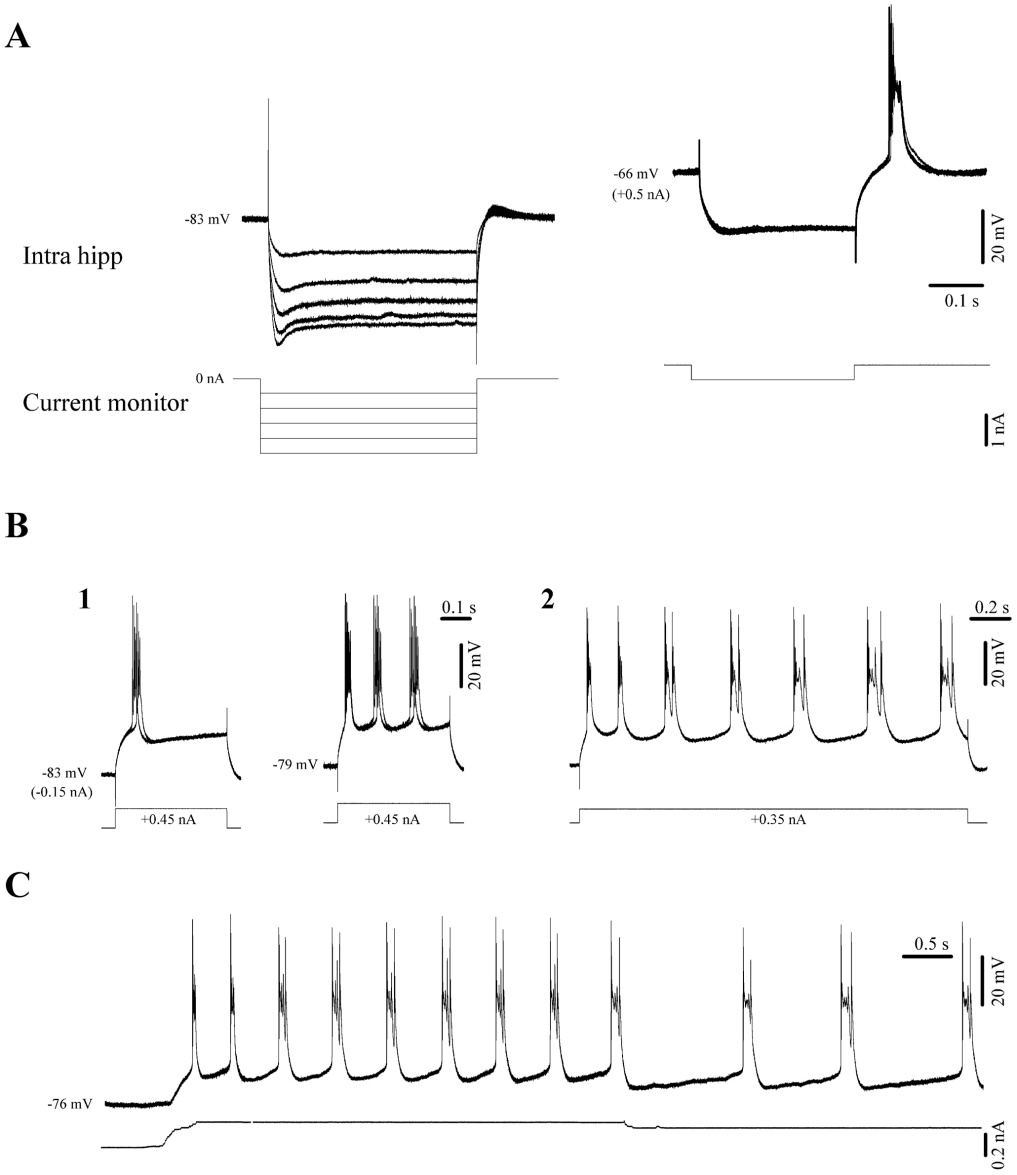
Intrinsic properties of CA3 neurons under isoflurane *in vivo*. (**A**) Hyperpolarizing pulses trigger I_h_ currents which increase with steady hyperpolarization (left). The same cell displayed discharge rebound bursts at more depolarized levels (right). (**B**) Depolarizing pulses trigger bursts of action potentials (**1**). Due to their activation characteristics we tentatively regard these bursts as high-threshold spikes (HTS). The HTS responses become rhythmic at more depolarized membrane potentials (**1** right). Controlling the cell’s membrane polarization modifies the frequency of these oscillations (**B2** and **C**).

In order to determine whether these events originate from self-sustained oscillations in hippocampal neurons and their intrinsic properties, we applied hyperpolarizing and depolarizing current pulses into the recorded cells. We found that during the νC state hippocampal neurons displayed I_h_ type currents (Fig. 5A left) and bursting capabilities (Fig. 5A right). Moreover, when steadily depolarized, hippocampal CA3 neurons spontaneously generated rhythmic bursts. The frequency of these bursts depended foremost on the cell’s membrane potential (Fig. 5B–C) – a finding that meshes well with earlier observations [23]. Thus, changes in the overall network excitatory/inhibitory balance and cellular input levels caused e.g. by anesthesia can modulate the oscillatory frequency of these neurons.

**Figure 6:**
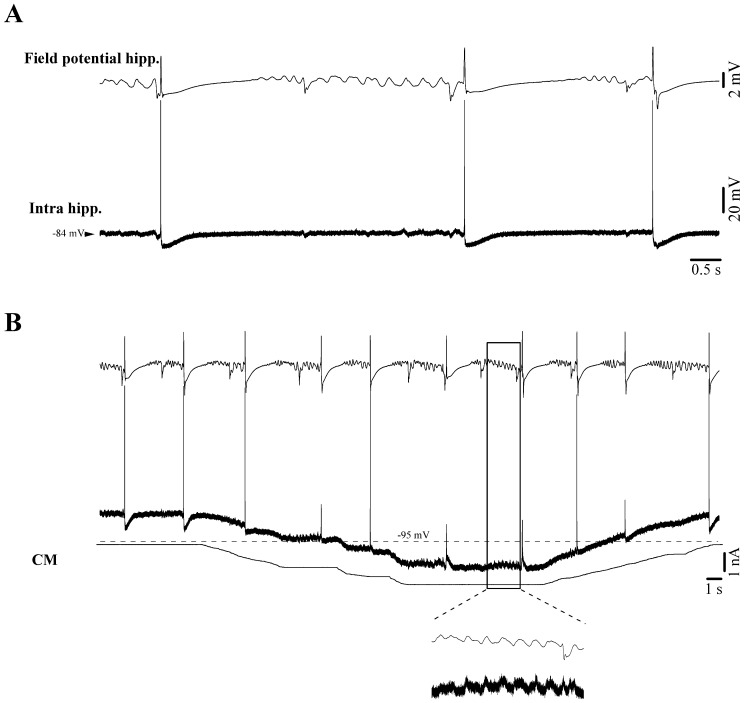
Intracellular recording of a putative dentate gyrus granule cell during νC state. (**A**) Under resting conditions the cell discharge bursts of action potentials in relation to νCs. Ripples displayed in the field potential recording (CA3 region) are not overtly paralleled by intracellular potentials. (**B**) Steady hyperpolarization of the neuronal membrane potential by intracellular current injection to very negative values (beyond −110 mV) reveals phasic events time-locked with the field potential delta ripples.

In addition to self-oscillating CA3–1 pyramidal cells, we recorded hippocampal neurons that displayed only minor EPSP responses during delta ripple activity (Fig. 6A), despite participating in νC events (*n* = 6 neurons). According to the stereotaxical coordinates, these neurons were situated in the dentate gyrus (DG). EPSPs related to delta ripples could only be fully revealed by significant hyperpolarization of the membrane potential (Fig. 6B), suggesting that delta ripples may have been insufficient in eliciting action potentials in these neurons. We presume these cells to be granule cells from the DG, which exhibit a very high excitability threshold due to the presence of an unusual set of synaptic as well as extrasynaptic non-desensitizing GABA_A_ receptors [24].

### Synchronization of Hippocampal Neurons during the NC State

**Figure 7:**
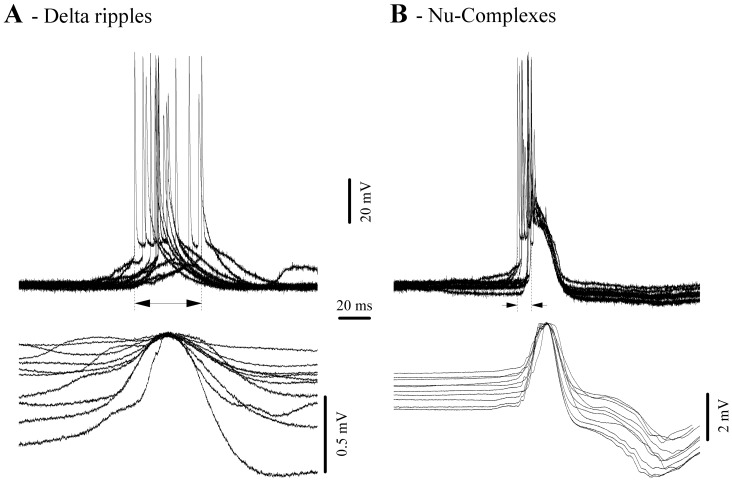
Synchrony of hippocampal neurons during delta ripples (A) and NCs (B). All traces are aligned on the maximum of the field potential. For each panel the vertical dotted lines indicate the earliest and the latest onset of neuronal action potential discharge, whilst the horizontal arrow depicts the dispersion of this onset and represents a measure of the degree of synchrony of hippocampal neurons.

Synchronization is a critical factor for the transmission of hippocampal activity towards the neocortex. Generally, the amplitude of field potentials is considered to reflect the number of synaptic inputs simultaneously contributing to the coherence of a particular pattern of activity. In the case of delta ripples we assessed the synchronization by measuring the jitter of the onset of neuronal discharge (this onset reflects the point where synaptic inputs from other neurons impose a significant level of depolarization) with respect to the maximum of the positive field potential wave associated with delta ripples (Fig. 7A). Depicted in Fig. 7A are 10 delta ripples with a dispersion of the first action potential over 44 ms. In the same hippocampal neuron, the equivalent jitter measured for NCs was of only 10 ms (Fig. 7B). After performing this comparison in all 39 recorded hippocampal neurons we obtained average jitters of 49.7 ms (±4.8) for delta ripples and 12.4 ms (±1.7) for NCs. The two sets of values were significantly different (signed-rank Wilcoxon test; p<0.001). We conclude that the hippocampal networks display greater synchronization during NCs than during delta ripples. This fact may play an important role within the scenario of genesis for NCs and their subsequent hippocampal-neocortical relay of NCs (see below).

## Discussion

### Scenario for the Genesis of νCs

**Figure 8:**
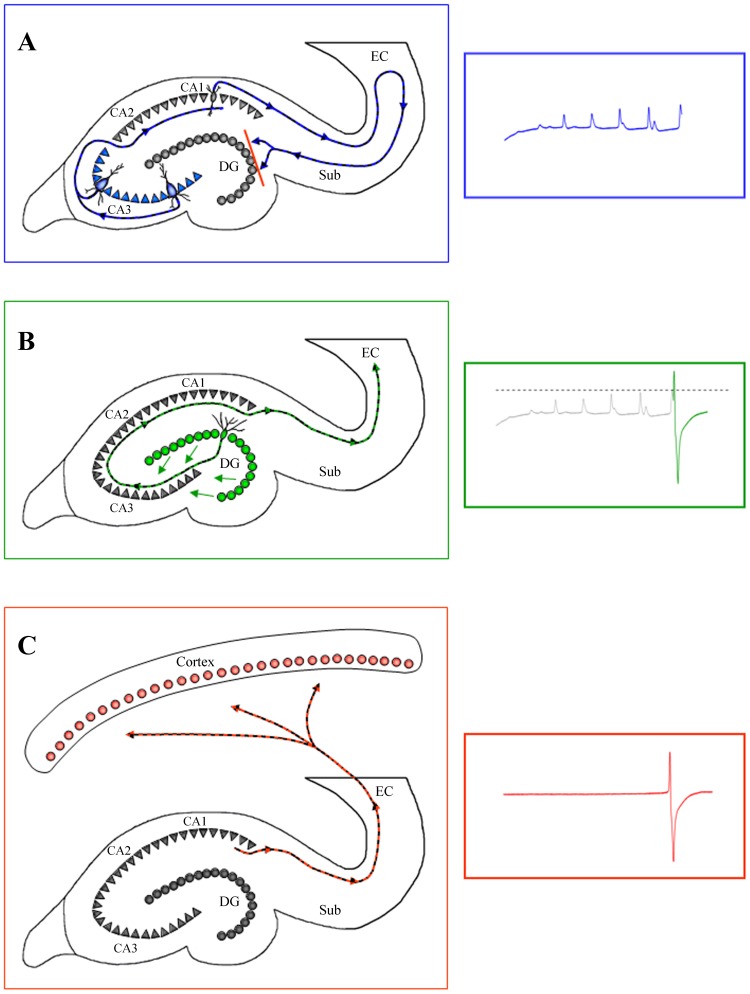
Schematic for the generation of νCs. (**A**) Generation of recurrent delta ripples within hippocampal neurons fails to activate the dentate gyrus. At right: hippocampal field potential activity. (**B**) Increased synchronization of delta ripples activates dentate gyrus granule cells, which in turn recruit hippocampal neurons, creating a νC. At right, entorhinal cortex field potential activities (green). (**C**) The newly generated νC activates cortically-projecting deep layers of the entorhinal cortex, permitting the propagation of the νC toward the neocortex. At right, scalp EEG activity.

From the above data we propose the following scenario to explain how self-oscillations of a limited population of CA3 pyramidal cells can lead to an activity spreading all throughout the brain (Fig. 8). To begin with, hippocampal CA3 neurons have long been known for their tendency to produce highly synchronized population bursts as well as self-sustained rhythmic activity [19], [25] due to their intrinsic membrane properties [23] and local inhibitory mechanisms [26]. Under normal conditions hippocampal CA3 neurons receive finely tuned excitatory and inhibitory inputs from subcortical structures, such as the medial septal nucleus and the diagonal band of Broca [27] generating specific rhythms such as the theta rhythm during exploratory behavior in rats and REM sleep [28]. Thus these neurons are usually under dominant control from afferent inputs, preventing the expression of intrinsically generated firing patterns [29]. The extent of this tightly controlled influence becomes apparent in isolated hippocampal preparations where highly synchronous population bursts are the predominant activity pattern [30]. During anesthesia-induced sleep-like patterns and further during BS, control through afferent signaling gradually diminishes, permitting the onset of self-oscillatory activity in CA3 pyramidal neurons [31]. At first, individual cells may follow only their own rhythm; however, through intra-hippocampal connections neighboring cells synchronize their activity and form local “hot-spots” of synchronized activity. Synchronization may also be aided by ephaptic (non-synaptic) processes i.e. activation of the neighboring cells through changes in the extracellular field when one neuron discharges [32], [33], although the only pertinent references stem from studies performed in slices. The increase in synchrony can be observed as an increase in amplitudes of local field potentials of ripple activity during increasing application of isoflurane (Figs. 3B1–4 and 8A). Impulses during ripple events travel along the intra-hippocampal loop, similarly to the well studied theta activity during exploratory behaviors. Thus, activity is passed on from the CA3 to CA1 region and from there through the subiculum, to the entorhinal cortex (EC), which presents the main output structure of the hippocampal formation [19]. Deep layers V–VI of the EC project towards cortical targets, whilst superficial layers I–III, send impulses back into the hippocampal loop via the perforant pathway towards the DG [31]. Each of the two populations of EC cells has a specific preferred input frequency: superficial layers I–III respond to activities within the theta range, whilst layers V–VI respond to fast and highly synchronized (140–200 Hz) frequencies such as in sharp waves [34]. Therefore, marginally synchronized ripple events will not be relayed to the cortex by the EC but will be directed towards the hippocampal DG via the perforant pathway. As mentioned above, DG granule cells show very low excitability and have been described as “gate” or “filter” for hippocampal afferent signals [35], [36]. Thus the relatively unsynchronized EPSPs of ripples are filtered out by granule cells and their propagation within the hippocampus stops at the DG gate (Fig. 8A). However, since the intra-hippocampal synchrony steadily increases in successive ripple events, the continuous excitation of the granule cell gate by ever increasing volleys leads to a decreasing activation threshold, as granule cells appear to be highly sensitive to preceding signaling activity [37]. Also, CA3 synchronization may have to undergo a certain threshold behavior before single-cell bursting activity leads to a truly synchronous population burst [38]. When this threshold is reached, DG activation is achieved. The divergent connectivity of granule cells [39] ensures their synchronous activation, which in turn results in highly concurrent propagation of the signal towards the rest of the hippocampus via the CA3: a ν-complex is generated (Fig. 8B). This event is then carried through CA2, CA1 and subiculum further towards the EC, where it now activates the deep V–VI layers instead of superficial layers due to the νC’s high frequency signal. Consequently, the νC event is further directed towards neocortical targets (Fig. 8C) from where it spreads into virtually the whole brain. The propagation of the same event backwards to the hippocampus, or a reverberation of the signal within the hippocampal loop is prevented, as massive population bursts such as νCs result in the activation of strong afterhyperpolarizations in hippocampal principal neurons including granule cells (lasting several hundred milliseconds) shunting the production of further action potentials [26]. This afterhyperpolarization is partly due to intrinsic neuronal properties, but is also a result of the inevitable triggering of local inhibitory networks by synchronized population bursts such as sharp waves [19] or νCs. Thus, a phase of silence follows each νC. During this period formerly synchronized CA3 pyramidal neurons lose their collective rhythm and ripple activity is re-set for a new cycle.

### Functional Implications of the Novel ν-complex State

In this paper we report an active brain state that extends well beyond deep coma associated with an EEG isoelectric line and potentially represents a new frontier in brain functioning. We have shown that νCs arise in the hippocampus and are subsequently transmitted to the cortex. The genesis of a hippocampal νC depends upon another hippocampal activity, known as ripple activity, which is not overtly detectable at the cortical level. The νC state is possible due to the intense subcortical activity generated in the hippocampus under conditions where cortical spontaneous functioning is greatly reduced. These observations have far-reaching consequences:

1) Although in this study the νC state was achieved using the volatile anesthetic isoflurane, the progression from wakefulness to BS and the isoelectric line is similar to other etiologies, anesthesia-induced or not, as is shown by the human data which triggered the subsequent experiments. The two main reasons for employing isoflurane in our study are: (a) the excellent level of control over anesthesia, and (b) its quick reversibility, allowing easy switching between various states. Isoflurane is a lipophilic agent [40] which decreases cerebral metabolism [41] and constitutes one of the most widely employed clinical anesthetic due to characteristics such as low depression of cardiac contractility, diminished heart rate and arterial pressure, as well as negligible liver or kidney toxicity [42]. These attributes may have greatly aided our investigation of the νC state in cats since high concentrations of isoflurane (up to 4%) did neither compromise vital functions (cardiac, respiratory, etc.), nor induce cell death in the brain.

Therefore, the νC state represents the deepest form of coma obtained so far, and demonstrates that the brain may remain operational beyond the EEG isoelectric line. However, in many clinical situations, the brain might cease to operate due to anoxic or toxic insults compromising neuronal integrity itself. Our results indicate that if the integrity of neuronal elements is preserved, then the brain may survive. Moreover, the discovery of this novel brain state and its underlying mechanisms draw attention to the difficulties in establishing clinical brain death and could thus revive discussions about the usefulness of depth recordings as an additional assessment criteria for brain death, as suggested by Walker in 1977 [43], to establish the irreversibility of brain damage not only from the scalp level.

At the very least the current findings should serve clinicians in their assessment of patients’ depth of coma in case they encounter EEG activity patterns indicative of the νC state. If these patterns were observed, it would be highly advisable to review the patient’s medication-regime with regards to coma-deepening drugs. Even though the νC state in our animal studies was fully reversible due to the use of isoflurane anesthesia, other underlying etiologies may be less safe if combined with medication. Therefore, the description and exploration of this phenomenon is potentially life saving.

2) In our view, the progression toward the νC state emphasizes the following concept related to brain mechanisms: wakefulness, as a state that hosts conscious processes and the domination of willful action is characterized by a predominance of neocortical activity. As these functions fade at the onset of unconsciousness, the orchestrating powers are relinquished to more basic structures such as the thalamus (in the case of sleep) or the limbic system (present data). When these structures are released from neocortical influence, they begin to pursue activity patterns on their own and proceed to impose these patterns on other brain regions including the neocortex.

Most of these activity patterns are already present throughout consciousness and unconsciousness. For example, hippocampal oscillations in the theta range in rodents are associated with sensory processing and the control of exploratory behaviors [44]. In our preparation, hippocampal theta oscillations were present during transient isoelectric episodes of BS. The oscillatory frequency then continuously decreased in parallel with the deepening of the coma. This was further paralleled by the slowing of intrinsically generated oscillations as a function of membrane polarization. Thus, the oscillatory frequency is not simply switched from one particular predetermined frequency band (e.g. theta) to another (e.g. delta) but rather displays a continuous evolution modulated by the depth of coma.

3) The presence of these oscillatory activities in the hippocampus raises some intriguing questions as to their possible involvement in mechanisms of plasticity related to learning and memory processes. The preparation itself and the easy reversibility from νC coma may prove particularly suitable for the testing of the role of hippocampal ripples either in their triggering of sharp waves as a mechanism of reinforcing memory circuits [31], or in downsizing the strength of neuronal connectivity for the purpose of synaptic homeostasis [45].
